# External immunity in ant societies: sociality and colony size do not predict investment in antimicrobials

**DOI:** 10.1098/rsos.171332

**Published:** 2018-02-07

**Authors:** Clint A. Penick, Omar Halawani, Bria Pearson, Stephanie Mathews, Margarita M. López-Uribe, Robert R. Dunn, Adrian A. Smith

**Affiliations:** 1 The Biomimicry Center, Arizona State University, Tempe, AZ 85287, USA; 2 Research & Collections, North Carolina Museum of Natural Sciences, Raleigh, NC 27601, USA; 3 Department of Biological Sciences, North Carolina State University, Raleigh, NC 27695, USA; 4 Biological Sciences, Campbell University, Buies Creek, NC 27506, USA; 5 Department of Entomology, Center for Pollinator Research, Pennsylvania State University, University Park, PA 16802, USA; 6 Department of Applied Ecology, North Carolina State University, Raleigh, NC 27695, USA; 7 Center for Macroecology, Evolution and Climate, University of Copenhagen, Copenhagen, 2100 Denmark

**Keywords:** antimicrobial secretions, antibiotics, social immunity, social insects, ants, entomopathogens

## Abstract

Social insects live in dense groups with a high probability of disease transmission and have therefore faced strong pressures to develop defences against pathogens. For this reason, social insects have been hypothesized to invest in antimicrobial secretions as a mechanism of external immunity to prevent the spread of disease. However, empirical studies linking the evolution of sociality with increased investment in antimicrobials have been relatively few. Here we quantify the strength of antimicrobial secretions among 20 ant species that cover a broad spectrum of ant diversity and colony sizes. We extracted external compounds from ant workers to test whether they inhibited the growth of the bacterium *Staphylococcus epidermidis*. Because all ant species are highly social, we predicted that all species would exhibit some antimicrobial activity and that species that form the largest colonies would exhibit the strongest antimicrobial response. Our comparative approach revealed that strong surface antimicrobials are common to particular ant clades, but 40% of species exhibited no antimicrobial activity at all. We also found no correlation between antimicrobial activity and colony size. Rather than relying on antimicrobial secretions as external immunity to control pathogen spread, many ant species have probably developed alternative strategies to defend against disease pressure.

## 1. Introduction

Pathogens and parasites exert strong selective pressures on social animals due to the dense living conditions of social animals and the high genetic relatedness among group members. In response, social species have evolved numerous strategies to combat pathogen spread [1]. In addition to individual immune responses [2–4], social species employ public health strategies to stop the spread of pathogens before they become prevalent. In social insects, these strategies represent a form of external immunity that includes grooming behaviours [5] and the secretion of antimicrobial compounds whose function is akin to our antibiotics [6]. Because of the production of these antimicrobial compounds, social insects have been identified as promising sources of new and diverse antibiotics [7–10]. Yet general theory linking the evolution of sociality with increased investment in antimicrobials has been empirically tested in relatively few insect taxa [11–13]. Without such theory, it is difficult to predict the subset of social insect species that are likely to prove most fruitful as study organisms in the search for new antibiotics.

Comparisons among social insect species have found that, in line with predictions, social species tend to produce stronger antimicrobials than do their solitary counterparts. A comparison of six bee species found that extracts from two semi-social and two fully social species had much stronger antimicrobial activity than did extracts from two solitary species [12]. Similarly, a comparison of nine wasp species found that two social species produced stronger antimicrobials than did solitary or communal nesting species [11]. Among social species, selection for stronger antimicrobials is also predicted to increase in those that form larger colonies, as dense living conditions are predicted to increase pathogen spread among individuals [14]. This prediction was supported in at least one taxon where a comparison of antimicrobial activity among eight species of thrips found that solitary species yielded no inhibitory compounds while the strongest antimicrobials were produced by species that live in the largest social groups [13]. The cumulative evidence from these studies suggests that investment in strong antimicrobials has evolved repeatedly in social lineages and may be particularly important for species that live in large, complex societies.

Unlike wasps, bees or thrips, in which some species are social and others solitary, all free-living ant species are highly social [15] and are therefore predicted to invest in strong antimicrobials. Ants are susceptible to a variety of fungal and bacterial pathogens [16–18], and inhabit environments in which both fungi and bacteria are abundant and diverse, such as soil and leaf-litter [19]. The ancestor to all modern ants possessed a special pair of glands, the metapleural glands, which when assayed in a number of ant species were found to secrete antimicrobial compounds effective against bacteria, fungi and yeasts [20–22]. The presence of the metapleural glands in early ant lineages has been suspected to have facilitated the dominance of ants in soil habitats [20]. Although some modern ant lineages have lost the metapleural glands (e.g. ants from the genera *Camponotus*, *Polyrhachis* and *Oecophylla* [23]), several of these same ant lineages appear to have evolved the ability to produce antimicrobial compounds via other glands. In particular, the venom gland has been identified as a source of compounds that are effective against entomopathogenic fungi and bacteria [24,25]. Identification of antimicrobial compounds from a handful of well-studied ant species has provided further support for the assertion that ants rely on antimicrobial secretions for pathogen defence. Whether all or most ant species produce antimicrobials, however, is not known.

Here, we test how antimicrobial investment differs among ant species that vary in colony size, and we place our findings within the context of previous studies testing antimicrobial strength in insects. We quantified the inhibitory effect of ant secretions on the growth of the bacterium *Staphylococcus epidermidis*. Testing inhibition of *Staphylococcus* bacteria is the standard technique to measure antimicrobial activity of insect compounds, and it is the same approach used for the comparative studies in insect groups described above [11–13,26,27]. In the long run, testing ant extracts against a variety of microbial taxa, including known entomopathogens, will be necessary to evaluate their full antimicrobial strength. Yet, because so little is known about the pathogens that are most likely to affect ant species, particularly bacterial pathogens, we take the conservative approach of considering bacteria that have been studied in other insect systems. Doing so offers us a common benchmark to compare the strength of antimicrobial compounds across taxa and to evaluate current theory. Based on previous studies on other insect taxa, we predicted that all or most ant species would invest in strong antimicrobials similar to other social insect taxa. We then predicted that ant species that form large colonies would invest in stronger antimicrobials than species that live in small colonies due to the higher risk of disease transmission within large groups. We incorporate worker body size and phylogenetic factors into our models.

## 2. Methods

### 2.1. Ant collection and extraction

**Figure 1.**
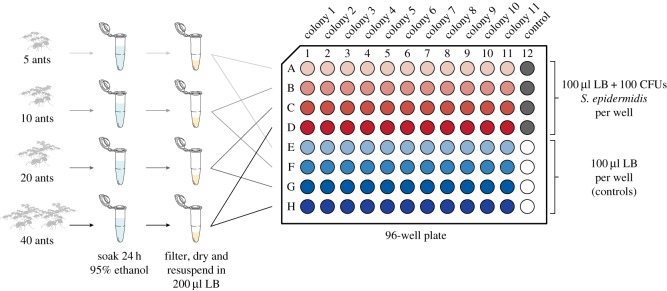
Extraction of ant surface compounds and microbial growth inhibition assay. Groups of ant workers (5, 10, 20 and 40) were soaked in 95% ethanol for 24 h. The ethanol was then pulled off, filter-sterilized and dried using an evaporator centrifuge. The dried extract was resuspended in 200 µl of LB. Half of the resuspended extract (100 µl) was added to a well in a 96-well plate containing 100 CFUs of *Staphyloccocus epidermidis* suspended in LB (100 µl), and the other half of the extract was added to a control well containing LB only (100 µl). Each column contained a sample from one colony, and the last column of each plate was reserved for controls containing either 100 CFU's of *S. epidermidis* in LB (rows A–D) or LB only (rows E–H). An additional 100 µl of LB was added to each well in the last column so that all wells contained a total of 200 µl of solution.

We compared antimicrobial strength of external body extracts from workers of 20 ant species collected in and around Raleigh, NC (USA) between May and October of 2016 (electronic supplementary material, table S1). The species we sampled covered a broad spectrum of ant diversity with members representing 18 genera from the four major ant subfamilies (Dolichoderinae, Formicinae, Myrmicinae and Ponerinae), and species-specific average mature colony sizes that varied from 80 to 220,000 individuals (electronic supplementary material, table S1). Care was taken to avoid sampling incipient colonies, and workers were collected from the periphery of mature nests or just inside mature subterranean nests. Workers were taken back to the laboratory, provided with water, separated from any soil that accompanied their collection and extracted within 24 h of collection. To extract putative antimicrobial compounds, we sampled workers from five different colonies per species. To test different concentrations of external compounds, we extracted groups of 5, 10, 20 and 40 workers from each colony in 95% ethanol for 24 h (figure 1). The ethanol extracts were then filter-sterilized (0.2 µm pore size polyethersulfone syringe filter), evaporated using an evaporator centrifuge (Vacufuge™, Eppendorf, Hamburg Germany) and resuspended in 200 µl of Lennox lysogeny broth (LB) (10 g l^−1^ tryptone, 5 g l^−1^ yeast extract and 5 g l^−1^ NaCl) for antimicrobial testing. We retained voucher specimens from each ant colony for species identification and body size measurements. We estimated body surface area of individual ant workers following the methods of Stow *et al*. [12].

### 2.2. Antimicrobial assay

To assess the antimicrobial strength of ant extracts, we tested whether they inhibited the growth of *S. epidermidis* in liquid culture. Using a 96-well plate, we added 100 µl of LB containing 100 colony-forming units (CFUs) of *S. epidermidis* to each well in the top four rows (we verified CFU number by plating nine serial dilutions (1 : 100) of *S. epidermidis* from LB liquid culture and comparing colony counts to optical density (OD) readings (*r*^2^ = 0.95)). To each well in the bottom four rows of the plate, we added 100 µl of LB only. We then divided each ant extract in half by adding 100 µl to a well containing *S. epidermidis* (test wells) and 100 µl to a well containing LB only (sterility controls). No ant extract was added to wells in the last column of each plate, which served as a positive control for *S. epidermidis* growth and a sterility control for LB (figure 1).

We quantified *S. epidermidis* growth based on the OD of each well at a wavelength of 590 nm using a microplate reader (SpectraMax^®^ M5, Molecular Devices, CA, USA). Plates were incubated at 37°C, and OD readings were taken after the plates were shaken at 1 h intervals over 24 h. Antimicrobial activity was quantified as an all or none response (electronic supplementary material, figure S1, S2); if the change in OD from the first to last reading was less than 10% of the change in OD from positive control wells, then we considered this complete inhibition. Inhibition was calculated using the following equation:

$$\Delta \text{OD}<0.1(\text{O}{\text{D}}_{\mathrm{final}}-\text{O}{\text{D}}_{\mathrm{initial}}),$$

where $\text{O}{\text{D}}_{\mathrm{initial}}$ is the OD of positive control wells at time zero, and $\text{O}{\text{D}}_{\mathrm{final}}$ is the OD at 24 h. We excluded samples if growth occurred in sterility control wells or if no growth occurred in positive controls.

### 2.3. Statistical analyses

To account for the non-independent evolutionary history among the lineages included in our dataset, we incorporated the phylogenetic distance between lineages as a random effect in a multivariate generalized linear mixed model (GLMM). We used the maximum likelihood phylogenetic tree of ants from Moreau & Bell ([28]; Tr66754 from TreeBASE) based on five nuclear genes (18S, 28S, abdominal-A, long-wavelength rhodopsin and wingless). If sequences for the species included in our study were not included in the phylogeny, we used the sequence from the closest relative (see raw data). To test for the effect of colony size on antimicrobial strength while correcting for body size, we incorporated log-transformed colony size and body surface as predictor variables in the GLMMs. Antimicrobial strength was calculated as the mean of the proportion of extracts that showed bacterial inhibition divided by the number of ants used in the assay. We used phylogenetically corrected models to account for the non-independent evolutionary history of the ant lineages by including correlation structures for Brownian motion, Ornstein-Uhlenbeck and Pagel's lambda calculated in the R package ‘ape' [29]. Model selection was based on the corrected Akaike information criterion (AICc) comparing the likelihoods of the ordinary and three phylogenetically corrected models.

## 3. Results

**Figure 2.**
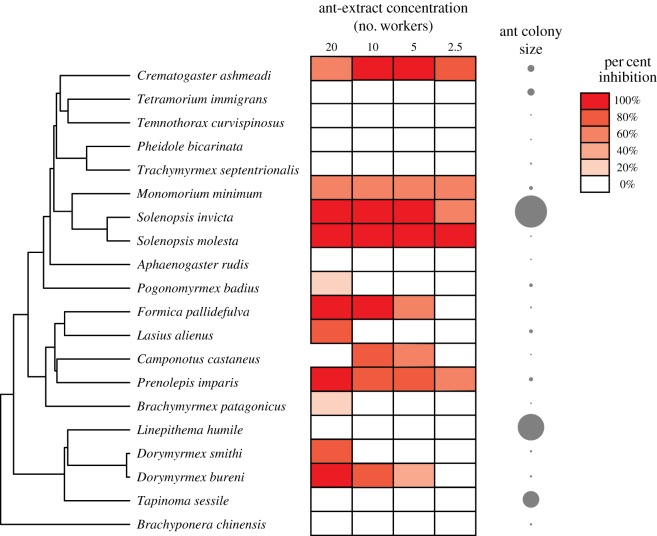
Antimicrobial inhibition of bacterial growth by extracts from 20 ant species. Species are grouped based on the phylogeny of Moreau & Bell [28]. Extract concentration varied by the number of ants extracted (highest concentration: 20 ants; lowest concentration: 2.5 ants). A relative comparison of colony sizes for each ant species is shown at right.

Based on previous research and theory, we predicted that extracts from all ant species would inhibit growth of *Staphylococcus epidermidis*. Extracts from over half of the ant species we tested inhibited bacterial growth (figure 2), but 40% did not. Even among the species for which inhibition was observed, the strength of antimicrobial activity varied. One-quarter of the species we tested inhibited *S. epidermidis* growth at the lowest ant-extract concentration, whereas other species showed inhibition only at very high ant-extract concentrations. The species exhibiting the strongest antimicrobial activity were broadly distributed across the ant phylogeny with one cluster in the tribe Solenopsidini (*Monomorium minimum, Solenopsis invicta* and *S. molesta*).

**Figure 3.**
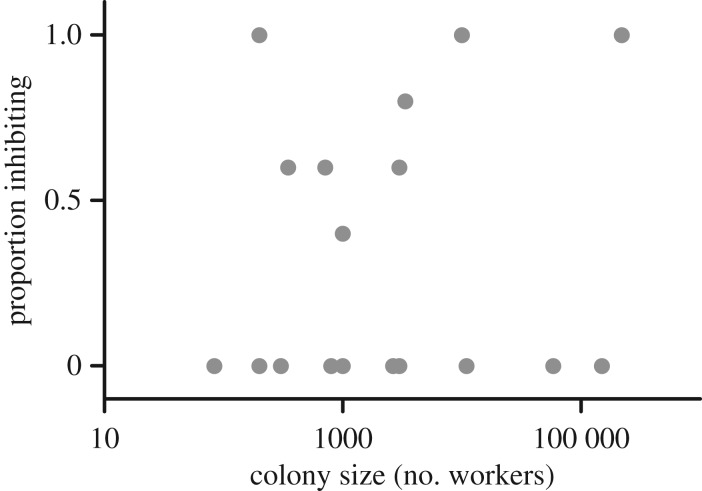
There were no significant relationships between antimicrobial strength and colony size using the GLM or Pagel's lambda models (*p* > 0.05) at any ant extract concentration (plot shows data from the 5-ant extract concentration).

We further predicted that species that live in large, dense colonies would produce stronger antimicrobials than species that live in smaller colonies. However, antimicrobial response was not predicted by colony size (*T = *0.5998; *p= *0.5565; table 1 and figure 3). Because ant species whose workers have a larger body size could theoretically produce and store larger quantities of potential antimicrobials per individual, we also included body size in our model but did not find a significant effect on antimicrobial production. To note, two of the species that exhibited the strongest antimicrobial activity in our study were among the smallest ants we tested (*Monomorium minimum* and *Solenopsis molesta*), and there was no clear pattern between body size and antimicrobial strength when compared together (electronic supplementary material, figure S3).

**Table 1.** Parameter estimates, statistical tests, and model comparison for ordinary and phylogenetically corrected models describing the relationship between antimicrobial strength and colony size.

Model	coefficient	s.e.	*t*	*p-*value	AICc	delta
ordinary	0.01689	0.0281	0.5998	0.5565	11.5	0
Brownian motion	0.00201	0.0240	0.0835	0.9344	14.0	2.42
Pagel's lambda	0.00946	0.0233	0.4048	0.6907	14.1	2.58
Ornstein-Uhlenbeck	0.01689	0.0281	0.5998	0.5565	15.2	3.62

## 4. Discussion

Social insects are thought to invest in strong antimicrobials for pathogen defence [12,30], but we found broad variation in antimicrobial investment among the ant species we tested. While past studies on individual ant species have identified potent antimicrobial compounds [6,7,22,26,31], a general comparison of antimicrobial investment across lineages has been lacking. In our comparison of 20 ant species representing 18 genera, 25% exhibited strong antimicrobial activity at a level comparable to that observed in social bees [12], social wasps [11] and social thrips [13]. However, 40% of the ant species we tested exhibited no detectable inhibition of *S. epidermidis* growth. This result is counter to our prediction that social species generally invest in strong antimicrobials, which was based on previous comparative studies of social insect antimicrobial inhibition of *Staphylococcus* bacteria. Instead, we found broad variation in antimicrobial investment among lineages. Clearly, not all social insect species invest in strong, general antimicrobials and must therefore use alternative means for pathogen defence.

Our analyses indicate that investment in general antimicrobials has evolved multiple times across the ant phylogeny (figure 2), but investment in antimicrobials seems to be independent of differences in colony size. Among social species, those that live in larger groups are predicted to invest in stronger antimicrobials than those that live in smaller groups. This prediction was supported by previous work on social thrips, which found higher antimicrobial activity in species that live in large colonies [13]. However, we found no relationship between colony size and microbial inhibition in ants. In addition to varying in colony size, the species we tested for antimicrobial activity varied 30-fold in body size (surface area, mm^2^). Large-bodied species have the potential to produce and store larger quantities of antimicrobial compounds, but we found no relationship between individual body size and antimicrobial strength in our assay. In fact, the species that exhibited the strongest antimicrobial activity in our study was the thief ant, *Solenopsis molesta*, which was among the smallest ants we tested and lives in some of the smallest colonies.

The primary source of antimicrobial secretions in ants has been thought to be the metapleural glands, which have been shown to inhibit the growth of bacteria and fungal pathogens in several ant species [7,22,32,33]. The few studies that have investigated major components of metapleural gland secretions identified phenols, carboxylic acids and fatty acids [21]. In this study, the source of the antimicrobial compounds in the raw extracts we tested are not known, but our use of a polar solvent would have extracted these known classes of active metapleural gland components. Antimicrobial compounds may have been produced by the metapleural glands of the ants we studied in some cases, but it in other cases they certainly were not. The metapleural glands have been lost in several ant lineages, including most species in the genus *Camponotus* [21]. Yet, we still observed antimicrobial activity in the species we tested from this genus (*C. castaneus*). Ants in the subfamily Formicinae, which includes *Camponotus*, produce formic acid as a defence compound against vertebrates and other insects, but formic acid is also used to inhibit fungal pathogens [34,35]. Similarly, the venoms of *Crematogaster* [36] and *Solenopsis* [8] are both known to inhibit the growth of *Staphylococcus* bacteria. Ants in the subfamily Dolichoderinae lack a stinging apparatus, but they produce defensive compounds in the pygidial gland that could also have antimicrobial properties [37,38]. Ant venoms, which can contain peptides and alkaloids, may be used as antimicrobials in those species that have a stinger [24,39], though not all. The ant in our study with the most potent venom against vertebrates, *Pogonomyrmex badius* [40], showed little antimicrobial activity.

Our assay tested the general strength of antimicrobial compounds against a common but non-pathogenic bacterium. Most known entomopathogens are fungal rather than bacterial, but most tests of antimicrobial secretions in ants have found broad-spectrum effects, including the inhibition of bacteria, fungi and yeasts [7,21]. Still, it is possible that ants have evolved specific antimicrobial defences that are activated against certain microbes but not others. Previous investigations of antimicrobial activity in social insects have found some variability in the action of antimicrobials on different microbes [8,10,22,41]. In fire ants, for example, antimicrobial venom compounds had stronger action against Gram-positive bacteria compared with Gram-negative [8]. For some species, the production of antimicrobials may be constant [42], but other species only produce antimicrobial compounds after exposure to certain pathogens. The termite *Reticulitermes flavipes*, for example, produces compounds that inhibit the growth of a broad range of human pathogens, but these compounds are only produced after termites are fed dead cells from those bacteria [10]. Social interactions may play a role as well. For example, leaf-cutting ants of the genus *Acromyrmex* use their metapleural glands to resist fungal infections [43], but this resistance is more effective when other ants are present to help groom and spread antimicrobials on infected individuals [44]. Some ant species may treat their nests with antimicrobials in addition to their bodies, such as ants in the genus *Formica*, which incorporate conifer resins into their nests to reduce bacterial and fungal loads [45–47].

A key finding from our study is that not all social insect species invest in strong antimicrobials. These species must therefore have developed alternative strategies to defend themselves against the higher exposure to pathogens associated with social living. One possibility is that, rather than producing compounds that directly kill pathogens, some ant species might produce compounds or have physical structures that promote the growth of beneficial microbes [48,49]. Cultivation of external defensive microbes has been documented sporadically across social and solitary insects [50]. In several genera of fungus-growing ants, workers cultivate Actinomycete bacteria on their bodies to treat parasitic fungal infections that attack their subterranean fungal gardens [51–53]. Similarly, *Coptotermes formosanus*, a subterranean termite, promotes the growth of Actinobacteria on their carton nests to help resist fungal entomopathogens [54]. Ants also host a diversity of endosymbiotic microbes, and similar to our findings for antimicrobial investment, certain ant lineages appear to be ‘hotspots' of microbial diversity, while others harbour very few microbial symbionts [55]. We hypothesize that the species in our assay that showed no evidence of producing antimicrobial surface compounds could be cultivating beneficial microbes that defend against pathogenic infection. Alternatively, species that showed little antimicrobial activity in our assay may rely on physical grooming or internal immunity to control pathogens [4].

In light of the rise in antibiotic resistant pathogens that infect an estimated 2 million people in the United States each year [56], research on pathogen control in social insects could provide future insights for dealing with antibiotic resistance. As others have suggested [7–10], social insects could be promising sources for new antibiotics effective against microbes resistant to our existing pharmacopeia of antibiotics. There are literally tens of thousands of social insect species to test for antimicrobial activity, but testing species at random is not an ideal strategy. While we found no correlation between colony size and antimicrobial activity, there may be other life-history features that could help narrow the search for species that produce strong antimicrobials, such as geographic distribution (e.g. temperate versus tropical) or nesting habitat (e.g. arboreal versus ground nesting). Also noteworthy is that we do not yet understand how species such as *Solenopsis invicta*, which produce strong antimicrobials, are able to prevent antimicrobial resistance from developing in their colonies. Further work on how social insects control pathogens without creating resistance could offer alternatives to our own use of antibiotics.

## Supplementary Material

Supplement

## Supplementary Material

Data

## Data accessibility

All data are included in the online electronic supplementary material.
