# Supplementary material for: External immunity in ant societies: sociality and colony size do not predict investment in antimicrobials
Source: R Soc Open Sci. 2018 Feb 7;5(2):171332. doi: 10.1098/rsos.171332 (PMC5830739; doi:10.1098/rsos.171332)
Supplement: Supplement [file rsos171332supp1.pdf]

## Supplement

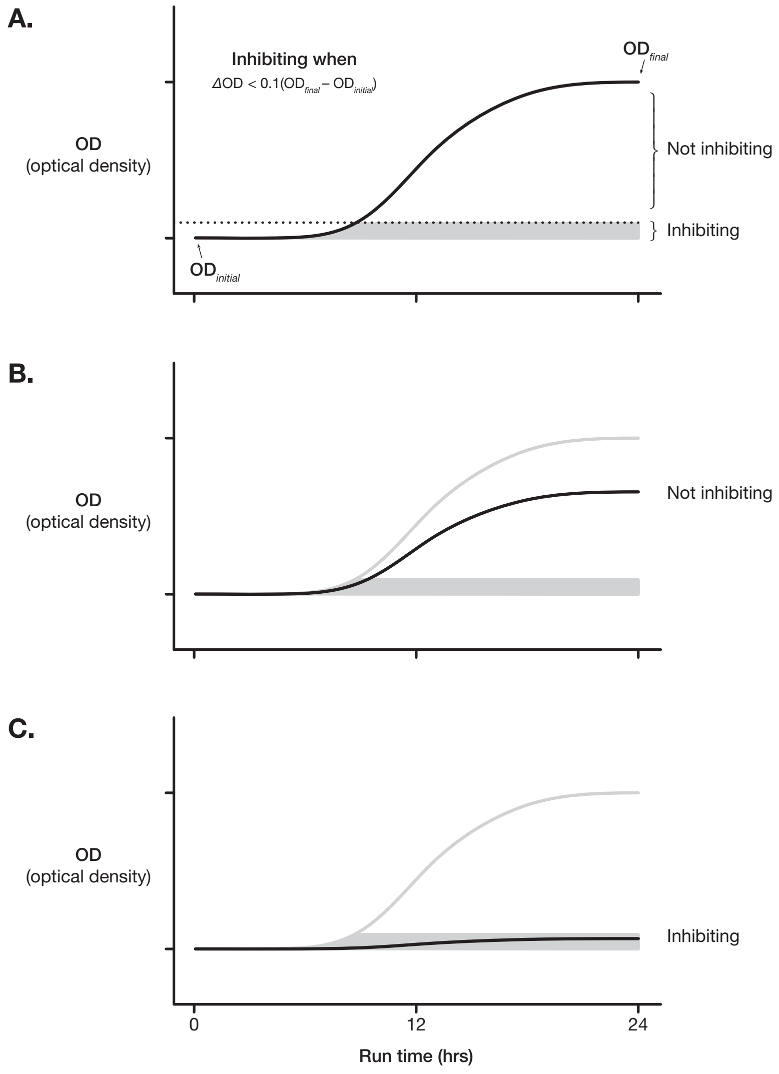


**Figure S1.** Quantification of microbial inhibition. (A) Growth of *S. epidermidis* in control wells measured as optical density (OD). Antimicrobial activity was quantified as an all or none response; if the change in OD from the first to last reading was less than 10% of the change in OD in positive control wells, then we considered this complete inhibition. Growth curves falling below the dotted line would be categorized as inhibiting *S. epidermidis* growth. (B) An example of a growth curve showing no inhibition. (C) An example of a growth curve showing inhibition.

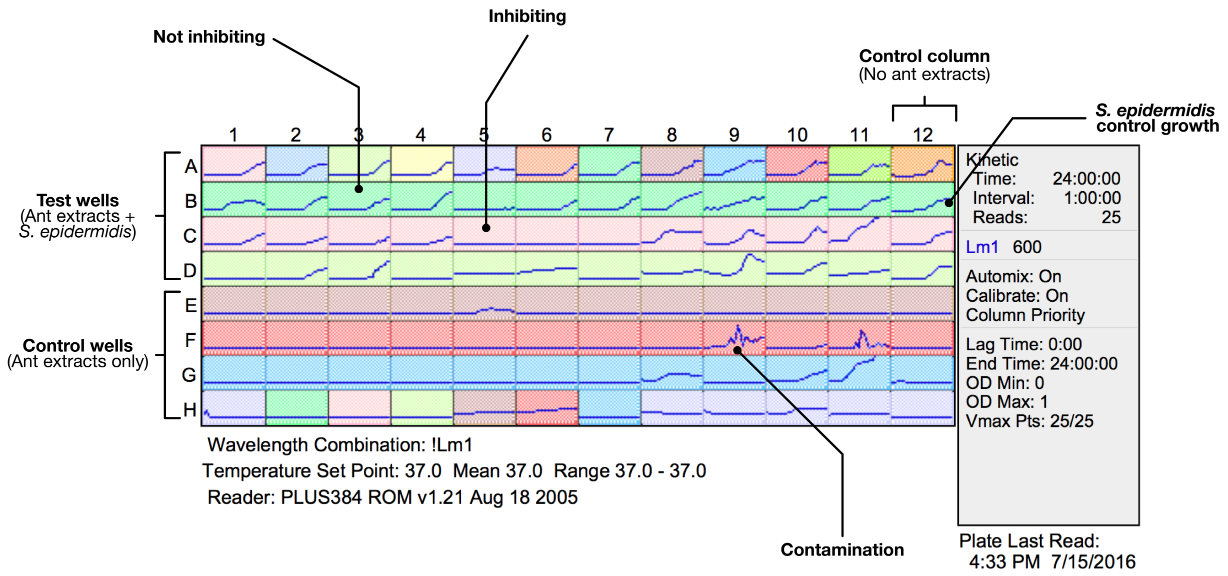


**Figure S2.** Example readout from plate reader. Top rows (A-D) include ant extracts plus *S. epidermidis*, while bottom rows (E-H) include ant extracts only to serve as a control. A flat line indicates no growth, which is common in ant extract control wells (rows E-H, columns 1-11). A flat line in test wells indicates inhibition. Standard growth curves for *S epidermidis* are found in column 12, rows A-D. Contamination is indicated by growth in ant extract control wells; if contamination was found in these rows, then we did not collect data from test wells and the assay was run again with fresh ant extracts. Several example wells are indicated in the figure above.

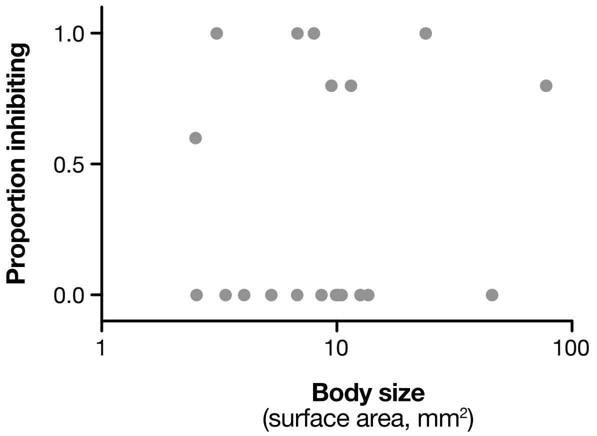


**Figure S3.** Variation in antimicrobial strength as a function of worker body size (surface area, $mm^2$).

**Table S1.** Ant collection information, body size, and colony size

| Species | Latitude | Longitude | Body size surface area (mm²)* | Colony size | Colony size references |
|---|---|---|---|---|---|
| *Aphaenogaster rudis* | 35.8122 | -78.7146 | 10.5 | 303 | Kaspari and Vargo 1995 |
| *Brachymyrmex patagonicus* | 35.7812 | -78.6528 | 2.5 | 200 | King and Porter 2007 |
| *Brachyponera chinensis* | 35.8122 | -78.7146 | 13.6 | 1000 | MacGown 2009 |
| *Camponotus castaneus* | 35.7872 | -78.6712 | 77.8 | 350 | King and Porter 2007 |
| *Crematogaster ashmeadi* | 35.7804 | -78.6391 | 6.8 | 10,000 | Tschinkel 2002 |
| *Dorymyrmex bureni* | 35.0645 | -79.5958 | 9.9 | 1,000 | King and Porter 2007 |
| *Dorymyrmex smithi* | 35.0645 | -79.5958 | 11.5 | 1,000 | Estimated based on *D. bureni* |
| *Formica pallidefulva* | 35.7872 | -78.6712 | 23.9 | 713 | Kaspari and Vargo 1995 |
| *Lasius alienus* | 35.7872 | -78.6712 | 8.6 | 3000 | Geraghty et al. 2007 |
| *Linepithema humile* | 35.7872 | -78.6712 | 5.3 | 150,000 | Beckers et al. 1989 |
| *Monomorium minimum* | 35.7872 | -78.6712 | 2.5 | 3,000 | Geraghty et al. 2007 |
| *Pheidole bicarinata* | 35.8122 | -78.7146 | 3.4 | 800 | Estimated from King and Porter 2007 |
| *Pogonomyrmex badius* | 35.0645 | -79.5958 | 45.8 | 2,658 | Tschinkel 2004 |
| *Prenolepis imparis* | 35.7812 | -78.6528 | 9.5 | 3,370 | Kaspari and Vargo 1995 |
| *Solenopsis invicta* | 35.7804 | -78.6391 | 8.0 | 220,000 | Kaspari and Vargo 1995 |
| *Solenopsis molesta* | 35.7804 | -78.6391 | 3.1 | 200 | King and Porter 2007 |
| *Tapinoma sessile* | 35.7812 | -78.6528 | 6.8 | 58,626 | Buczkowski 2010 |
| *Temnothorax curvispinosus* | 35.8805 | -78.7584 | 4.0 | 84 | Geraghty et al. 2007 |
| *Tetramorium Sp. E* | 35.7804 | -78.6391 | 10.1 | 10,975 | Kaspari and Vargo 1995 |
| *Trachymyrmex septentrionalis* | 35.8805 | -78.7584 | 12.6 | 800 | Kaspari and Vargo 1995 |

*Calculated as surface area of a cylinder using ant length and width measurements

## References


Beckers, R., S. Goss, J.-L. Deneubourg, and J. Pasteels. 1989 Colony size, communication, and ant foraging strategy. *Psyche* 96:239-256.

Buczkowski, G. 2010 Extreme life history plasticity and the evolution of invasive characteristics in a native ant. *Biological Invasions* 12:3343-3349.

Geraghty, M. J., R. Dunn, and N. J. Sanders. 2007 Body size, colony size, and range size in ants (Hymenoptera: Formicidae): Are patterns along elevational and latitudinal gradients consistent with Bergmann's rule. *Myrmecological News* 10:51-58.

Kaspari, M. and E. L. Vargo. 1995 Colony size as a buffer against seasonality: Bergmann's rule in social insects. *The American Naturalist* 145:610-632.

King, J. R. and S. D. Porter. 2007 Body size, colony size, abundance, and ecological impact of exotic ants in Florida's upland ecosystems. *Evolutionary Ecology Research* 9:757-774.

MacGown, J. 2009 The Asian Needle Ant, *Pachycondyla chinensis* (Emery)(Hymenoptera: Formicidae), Reported from Alabama. *Midsouth Entomologist* 2:88-89.

Tschinkel, W. R. 2002 The natural history of the arboreal ant, *Crematogaster ashmeadi*. Journal of Insect Science 2:1-15.

Tschinkel, W. R. 2004 The nest architecture of the Florida harvester ant, *Pogonomyrmex badius. Journal of Insect Science* 4:1-19.
